# Evaluating the vector competence of *Aedes simpsoni sl* from Kenyan coast for Ngari and Bunyamwera viruses

**DOI:** 10.1371/journal.pone.0253955

**Published:** 2021-07-01

**Authors:** James Mutisya, Michael Kahato, Francis Mulwa, Solomon Langat, Edith Chepkorir, Samuel Arum, David Tchouassi, Rosemary Sang, Joel Lutomiah

**Affiliations:** 1 Centre for Virus Research, Kenya Medical Research Institute, Nairobi, Kenya; 2 Institute of Tropical Medicine and Infectious Diseases, Jomo Kenyatta University of Agriculture and Technology, Nairobi, Kenya; 3 Human Health Department, International Centre of Insect Physiology and Ecology, Nairobi, Kenya; CEA, FRANCE

## Abstract

**Background:**

Bunyamwera(BUNV) and Ngari (NGIV) viruses are arboviruses of medical importance globally, the viruses are endemic in Africa, *Aedes(Ae) aegypti* and *Anopheles(An) gambiae* mosquitoes are currently competent vectors for BUNV and NGIV respectively. Both viruses have been isolated from humans and mosquitoes in various ecologies of Kenya. Understanding the risk patterns and spread of the viruses necessitate studies of vector competence in local vector population of *Ae*. *simpsoni sl* which is abundant in the coastal region. This study sought to assess the ability of *Ae*. *Simpsoni sl* mosquitoes abundant at the Coast of Kenya to transmit these viruses in experimental laboratory experiments.

**Methods:**

Field collected larvae/pupae of *Ae*. *Simpsoni sl* mosquitoes from Rabai, Kilifi County, were reared to adults, the first filial generation (F0) females’ mosquitoes were orally exposed to infectious blood meal with isolates of the viruses using the hemotek membrane feeder. The exposed mosquitoes were incubated under insectary conditions and sampled on day 7, 14 and 21days post infection to determine susceptibility to the virus infection using plaque assay.

**Results:**

A total of 379 (Bunyamwera virus 255 and Ngari virus 124) *Ae*. *simpsoni sl* were orally exposed to infectious blood meal. Overall, the infection rate (IR) for BUNV and NGIV were 2.7 and 0.9% respectively. Dissemination occurred in 5 out 7 mosquitoes with mid-gut infection for Bunyamwera virus and 1 out of 2 mosquitoes with mid-gut infection for Ngari virus. Further, the transmission was observed in 1 out of 5 mosquitoes that had disseminated infection and no transmission was observed for Ngari virus in all days post infection (dpi).

**Conclusion:**

Our study shows that *Ae*. *simpsoni sl*. is a laboratory competent vector for Bunyamwera virus since it was able to transmit the virus through capillary feeding while NGIV infection was restricted to midgut infection and disseminated infection, these finding adds information on the epidemiology of the viruses and vector control plan.

## Introduction

Bunyamwera virus (BUNV) and Ngari virus (NGIV) are negative-sense single stranded enveloped RNA viruses that belong to the genus *Orthobunyavirus* and family *Bunyaviridae* [1]. Bunyamwera virus and NGIV are widely distributed throughout large parts of Africa. In the sub-Saharan Africa, they mainly circulate in the forests in enzootic transmission cycles involving non-human primate hosts and forest dwelling mosquito vectors. The epizootic cycle involves birds, ruminants and mosquitoes; and epidemic cycle involves human and mosquito vectors [2]. Bunyamwera virus was first isolated in 1943 from *Aedes* mosquitoes in Uganda, as part of yellow fever surveillance in the Semiliki Forest [3]. Since then, the virus has been isolated in several other African countries including Kenya [4] from *Aedes ochraceus*, *Aedes mcintoshi* and *Anopheles funestus* mosquitoes. Evidence suggests *Ae*. *aegypti* might be the primary mosquito vector of BUNV [5, 6]. Experimental studies have shown that *Ae*. *aegypti* populations from Kenya are competent in transmitting BUNV, but not NGIV. However, *An*. *gambiae sl* was competent for both viruses, while *Culex quinquefasciatus* failed to transmit any of the two viruses [6]. A second strain of BUNV was isolated in 1955 from *Aedes circumluteolus* in Tongaland, South Africa [7]. In Kenya, the virus was isolated from Rift Valley Fever (RVF) mosquito positive samples collected from northeastern Kenya during RFV outbreak [8, 9]. However, the actual role of the associated mosquito species in the maintenance and transmission of the virus in the environment remains unclear. Bunyamwera virus has recently been associated with human disease outbreaks especially within the East African region [1, 10].

Ngari virus was first isolated from *Aedes simpsoni* in 1979 in Southeastern Senegal and later isolated from several other mosquito species in Burkina Faso, Central African Republic and Madagascar between 1988 and 1993 [11]. In Kenya, the virus was isolated from mosquitoes and humans with haemorrhagic fever symptoms during RVFV outbreak in Kenya and Somalia in 1998–1999 [1]. This outbreak was associated with approximately 89,000 human infections and over 250 deaths. Both viruses are etiological agents of diseases in humans and domestic animals. Bunyamwera virus is associated with mild symptoms, such as fever, joint pain, and rash in many mammals including humans [12, 13], while NGIV causes fatal hemorrhagic fevers in both humans and ruminants [1, 14]. Ngari virus is of particular public health relevance as it was identified as other etiologies of febrile illnesses in humans in Sudan, Somalia, and Kenya in 1988, 1997, and 1998, respectively [1, 8]. In West Africa, NGIV has been isolated from diverse mosquito species including *Anopheles gambiae*, *Anopheles pharoensis*, *Cxulex*. *antennatus*, *Culex poicilipes* and *Culex tritaeniorhynchus* [11, 15]. Ngari virus comprises of three segments small, medium and large segments. Reassortments are relatively common occurrence among viruses from the Bunyaviridae family and Ngari virus is a reassortant between BUNV and Batai virus (BATV) [10, 16], with the medium segment originating from BATV and the small and large segments from BUNV.

Ngari virus has been isolated in many mosquito vectors, such as *Aedes argenteopunctatus*, *Aedes minutus*, *Aedes vexans*, *Ae*. *mcintoshi*, *Anopheles coustani*, *Aedes neoafricanus*, *Ae*. *simpsoni*, *Ae*. *vittatus*, *Anopheles pretoriensis*, *Anopheles pharoensis*, *Anopheles mascarensis*, *Culex bitaeniorhynchus*, *Cx*. *tritaeniorhynchus*, *Culex antennatus* and *Cx*. *poicilipes* in Senegal and in Kenya [11, 17, 18]. These isolations suggest a wide vector range, which could indicate the potential for widespread geographic distributions as well as potential vertebrate host ranges, given the diversity of feeding preferences of these mosquito vectors. Thus, the burden of disease of NGIV and likely BUNV and BATV is underreported and their public health impact under-appreciated.

Vector competence of mosquitoes is affected both intrinsic and extrinsic factor that affects the ability of vectors to get infected, maintain and transmit the viruses [19–21]. While vector competence of coastal *Ae*. *aegypti* for BUNV and NGIV has been determined [6], no studies have been conducted with regard to coastal populations of *Ae*. *simpsoni sl*. The coastal region, which is a tourist destination, neighbors northeastern Kenya and Somalia. Increased transboundary travels between these regions increases the risk of cross border transmission of these viruses and potential for outbreaks. *Ae*. *simpsoni sl*. is abundant at the coastal region and demonstrated to be competent vectors of other disease-causing viruses such as Yellow fever virus [22] and Chikungunya virus [23]. This study determined the infection, dissemination and transmission potential of the two viruses by *Ae*. *simpsoni sl*. This finding will be crucial in decision making and policy implementation to aid in mitigating the outbreak of these viruses in disease prone areas.

## Materials and methods

### Study sites

**Fig 1:**
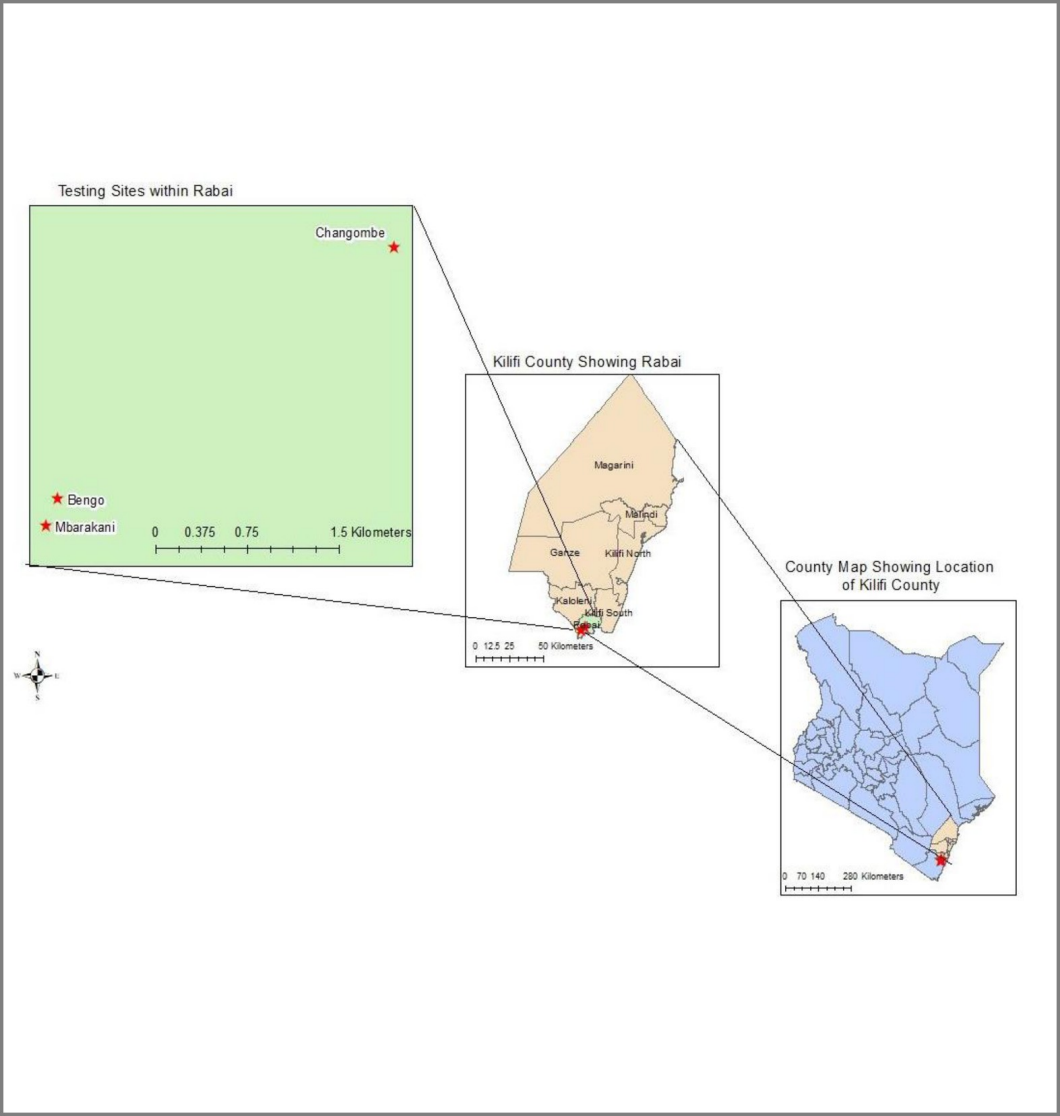
Map showing vector sampling sites in the coastal region of Kenya.

Mosquito larvae and pupae were collected from rural villages of Changombe, Mbarakani and Bengo in Rabai sub-County, Kilifi County, coastal region (Fig 1) using a larval sampling tool. Kilifi County has a bimodal pattern of rainfall with the long rains occurring from mid-April to end of June, with the highest rainfall occurring in the month of May. The short rains occur in November and December, and are generally unreliable. The County has annual mean temperature ranging from 21°C and 30°C in the coastal belt and 30°C and 34°C in the hinterland. The average annual rainfall ranges from 300mm in the hinterland to 1300mm at the coastal belt. The County experiences relatively low wind speeds ranging between 4.8 km/hr and 12 Km/hr. The main topography is coastal plains and island plains [24].

### Mosquito sampling

*Ae*. *simpsoni sl*. larvae and pupae were collected outdoors in peri-domestic areas on August 2017 using standard larval sampling tools in water holding containers in both artificial and natural breeding sites such as banana leaf axils, coconut and arrow roots. The collected larvae were transported to the KEMRI level 2 insectary for rearing to adults as described below.

### Mosquito rearing

Larvae were placed on larval trays and fed on Tetramin Fish food until pupation. The pupae were transferred in small cups containing water and placed in 4-liter plastic cages with netting material, for them to emerge to adults. The adults were knocked down at -20°C for 30 seconds and morphologically identified under a dissecting microscope to ensure that only *Ae*. *simpsoni sl*. were used in the study. The identified adult mosquitoes were returned to the 3-liter plastic cages and provided with 10% glucose solution *ad libitum* on cotton wool maintained at 28–32°C, 70–80% relative humidity and 12:12 hour light: dark (L:D) photoperiod.

### Virus amplification

The viruses used in this study were isolated during arbovirus surveillance activities in Kenya under the project: “Arbovirus Surveillance: Surrogate Epidemiologic Methods to Assess Arboviral Infection Distribution by Entomological Surveillance under KEMRI SSC # 824, BUNV isolate was isolated from *Ae*. *mcintoshi* sampled from Garissa (GSA/S4/11232) and NGIV was isolated from *An*. *funestus* from Tana Delta region (TND/S1/19801). The viruses were passaged in confluent monolayers of Vero (P12) cells in T-25 cell culture flasks, grown in Minimum Essential Medium (MEM), (Sigma-Aldrich, St. Louis, MO) with Earle’s salts and reduced NaHCO_3_, supplemented with 10% heat inactivated fetal bovine serum (FBS), (Sigma-Aldrich, St. Louis, MO), 2% L-glutamine (Sigma-Aldrich, St. Louis, MO), 2% antibiotic/ antimycotic solution with 10,000 units penicillin, 10 mg streptomycin and 25μg amphotericin B per ml (Sigma-Aldrich, St. Louis, MO). The inoculated monolayers were incubated at 37°C for 1 hour, to allow for virus adsorption. Maintenance medium (MEM with 2% FBS, 2% glutamine, 2% antibiotic/ antimycotic) was added, incubated at 37°C and 5% CO_2_ and observed daily for cytopathic effects (CPE). CPE for BUNV and NGIV was observed after 4 days and 6 days post inoculation, respectively. At 80% CPE, the flask was frozen overnight at -80°C, thawed on wet ice then clarified by centrifugation at 5000 revolutions per minutes and the supernatant harvested by aliquoting into 1.5 ml Cryovials and stored at -80° C until use.

### Virus quantification

Quantification of BUNV and NGIV were done by plaque assay. Ten-fold serial dilutions of amplified BUNV and NGIV was carried out and inoculated in 12-well plates containing confluent Vero cell monolayers grown in MEM, supplemented with 10% heat-inactivated FBS, 2% L-glutamine, 2% antibiotic/antimycotic solution and incubated at 37°C in 5% CO_2_ overnight. Each well was inoculated with 100 microliters of diluted virus, incubated for 1 hour with frequent rocking to allow adsorption. Infected cells were maintained using 2.5% methylcellulose mixed with 2X MEM and incubated at 37°C in 5% CO_2_. After 9 days, the plates were fixed for 1 hour with 10% formalin (Sigma), stained for 2 hours with 0.5% crystal violet (Sigma), washed, dried, and plaques were counted and calculated to quantify the virus as follows [25]:

$$\frac{Number\;of\;plaques}{d\times V}=pfu/ml$$

where **d** is the dilution factor and **V** is the volume of diluted virus added to the well.

### Oral infection of mosquito

Four-day old adult female *Aedes simpsoni sl*. were deprived of water and glucose for 12 hours before they were orally exposed to infectious blood meal mixed with either quantified BUNV (10^5.18^ PFU/ml) or NGIV (10^7.42^ PFU/ml) and defibrinated sheep blood at a ratio of 1:1. The exposure time was one hour, using parafilm as an artificial membrane feeding system (Hemotek), which employs an electric heating element that maintains the temperature of the blood meal at 37°C. After feeding, fully engorged mosquitoes were transferred in a clean 1litre -gallon plastic cage with a netting material and maintained with 10% glucose solution at a temperature of 28–32°C, relative humidity of 80% and 12:12 hour Light: Day photoperiod. Mortality was monitored on daily basis.

### Determination of infection and dissemination rates

On day 7, 14 and 21 post-infection, a proportion of mosquitoes were sampled from both sets of experiments (BUNV and NGIV) and tested for infection, dissemination and transmission, respectively. Each mosquito was dissected, and body and legs separated and put in individually clean 1.5 eppendorf tubes with 500 microliters homogenization media, consisting of MEM, supplemented with 15% FBS, 2% L-Glutamine, and 2% antibiotic/antimycotic. The individual body was homogenized using a Mini bead beater (BioSpec Products Inc, Bartlesville, OK 74005 USA) with the aid of a copper bead (BB-caliber airgun shot) and clarified by centrifugation at 12,000 rpm (Eppendorf centrifuge 5417R) for 10 minutes at 4°C and the supernatant inoculated in 12 well plates containing confluent Vero cells, grown in MEM, supplemented with 10% FBS, 2% L-Glutamine and 2% antibiotic/antimycotic. The infected cells were maintained using 2.5% methylcellulose mixed with 2X MEM with 2% FBS, 2% L-Glutamine and 2% antibiotic/ antimycotic and incubated at 37°C with 5% CO_2_ for 6 days depending on the virus, then fixed and stained as described above. Infection rate for BUNV or NGIV was determined as proportion of positive bodies over total orally exposed mosquitoes. For disseminated infection rates, only the legs of BUNV and NGIV positive bodies were homogenized and tested as described above. Presence of virus in the bodies and legs indicated successful infection and dissemination. The experiment was done in three replicates to ensure sufficient sample size for statistical analysis. Plaques were counted and values obtained were used to quantify the virus in the bodies and legs.

### Determination of transmission potential

To determine the transmission potential, individual mosquitoes were immobilized on 7,14 and 21 dpi by freezing in -20°C freezer for 40 seconds, the wings and legs removed, and the proboscis inserted into a capillary tube containing homogenization media (HM), made of MEM, supplemented with 15% FBS, 2% L-Glutamine, and 2% antibiotic/ antimycotic. After 30 min of salivation, HM containing saliva was expelled into a cryovial containing 200μl homogenization media and later inoculated in Vero cell lines grown in 24 well plates. The infected cells were maintained in MEM with 2% FBS, 2% L-Glutamine and 2% antibiotic/ antimycotic and incubated at 37°C in 5% CO_2_ incubator and CPE observed daily for 6 days.

### Statistical analysis

Infected mosquitoes were given a unique code with the area, site of collection and numbers of mosquito processed recorded and data entered in MS Excel database. Infection rate was determined for each virus as a percentage of mosquitoes with virus in the body out of the total exposed mosquitoes, dissemination rate as percentage of infected mosquitoes with virus in the legs out of the total of mosquitoes with midgut infection and transmission rate as percentage of mosquitoes with positive saliva out of the total disseminated infection that passes over the virus to the diluent via saliva by capillary feeding technique. The infection, dissemination and transmission rate were analyzed using Chi-square test. Tukey’s test was used to analyze the means of viral titers for BUNV and NGIV positive samples. The infection, dissemination and transmission data was presented by use of frequencies and percentages, and displayed on charts. Confidence interval was calculated at 95% based on binomial distribution. P-values equal or <0.05 was considered statistically significant. Dissemination efficiency was calculated as the number of disseminated infection divided by the number of individuals with midgut infection. Transmission efficiency was calculated as the number of infected saliva divided by the number of individuals with disseminated infection.

### Ethical considerations

Mosquitoes were fed on commercially obtained sheep blood from Kabete veterinary laboratories. Approval for implementation of this study was obtained from the KEMRI Scientific and Ethics Review Unit (KEMRI/SERU/CVR/012/3585).

## Results

### Infection, dissemination and transmission rates for BUNV and NGIV

A total of 379 *Ae*. *simpsoni sl* were orally exposed to NGIV (n = 124) and BUNV(n = 255). The mosquitoes were orally exposed to infected blood meals at titers of log10^5.18^ and 10^7.42^ PFU/ml for NGIV and BUNV, respectively. There was no difference in the infection rates of BUNV at 7 (3.9%, 4/102), 14 (1.1%, 1/89) and 21(3.1%, 2/69) days post infection (dpi) (*P*>0.5). For NGIV the midgut infection rates at 7 (4.8%, 2/42), 14 (0%, 0/42) and 21(0%, 0/40) dpi were lower compared to BUNV although not significantly (p>0.5). There was no significant difference in body infection between BUNV and NGIV on 7, 14 and 21 dpi as shown (Table 1). Virus dissemination occurred at 7- and 14- dpi for BUNV, with a total of 5 mosquitoes confirmed to have disseminated virus during the experiment. On the other hand, for NGIV, dissemination was observed only at 7 dpi, where virus dissemination was detected in one out of two mosquitoes with midgut infection. Although the proportion of BUNV-infected saliva from *Ae*. *simpsoni sl* was low, the transmission was still observed on 7 dpi. Overall, dissemination and transmission efficiency for Bunyawera were 2% and 0.4% respectively while Ngari virus had a dissemination efficiency of 0.8%.

**Table 1:** Infection, dissemination and transmission rates of BUNV and NGIV at 7, 14- and 21-days post-infection.

Viral strain	Days post-infection (dpi)	Number of individuals	% of infection	Dissemination	Transmission	Dissemination efficiency	Transmission efficiency
Bunyamwera	7	102	4 (4/102)	(4/4)	(1/4)	4 (4/102)	1 (1/102)
Ngari	7	42	5 (2/42)	(1/2)	(0/1)	2 (1/42)	-
Bunyamwera	14	89	1 (1/89)	(0/1)	-	-	-
Ngari	14	42	0 (0/42)	-	-	-	-
Bunyamwera	21	64	3 (2/64)	(1/2)	(0/1)	2 (1/62)	-
Ngari	21	40	0 (0/40)	-	-	-	-

- No test done.

Similarly, there was no significant difference observed for infection and transmission between two successive days post-infection for both the BUNV and NGIV.

**Fig 2:**
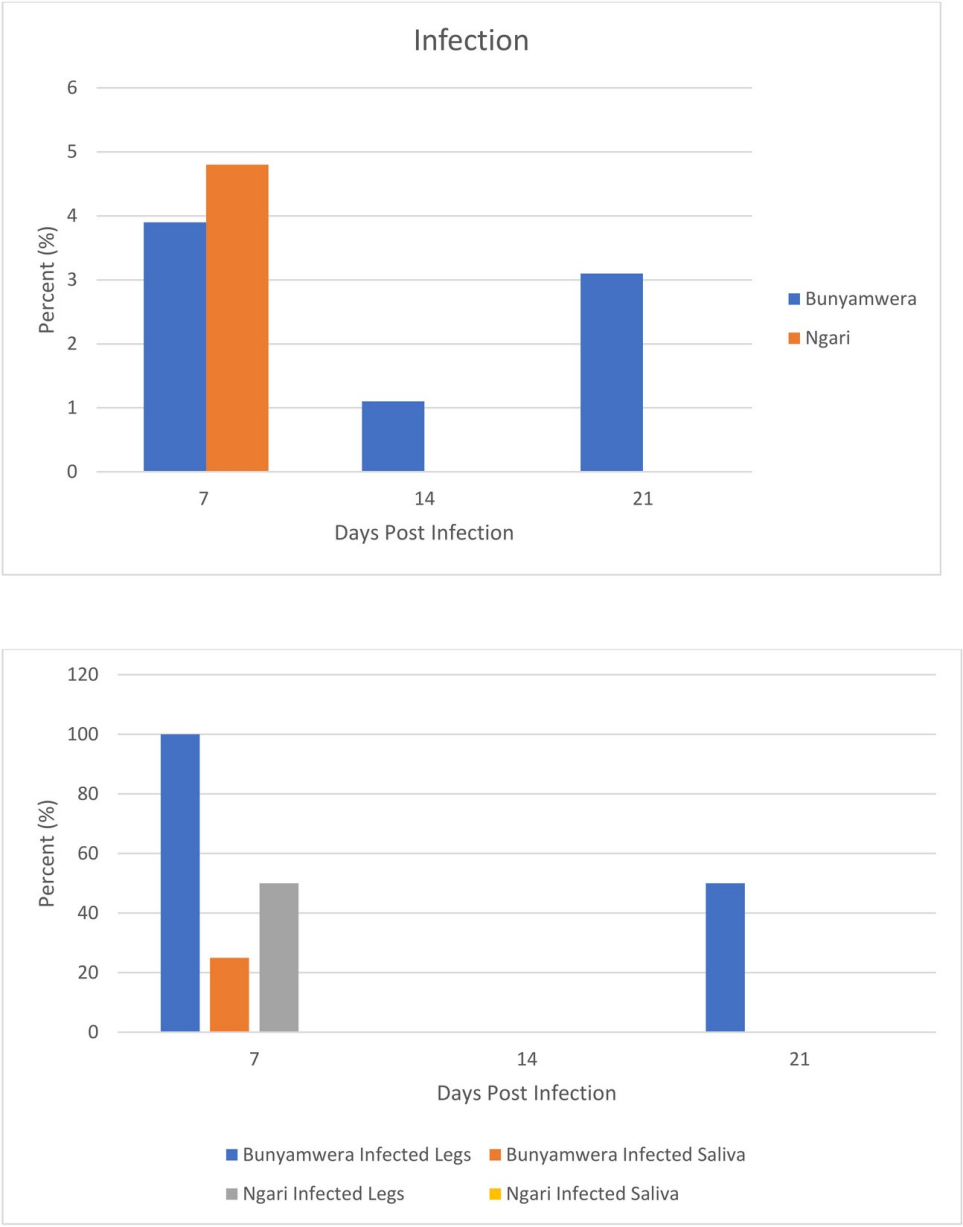
Infection rates (A), dissemination and transmission rates (B) of Bunyamwera and Ngari viruses in orally exposed *Ae*. *simpsoni sl*.

*Aedes*. *simpsoni sl*. showed strong midgut infection barrier for the two viruses with low midgut infection rate of 1.6% and 2.7% observed for NGIV and BUNV, respectively. There was no significant difference in their susceptibility to the two viruses (Tukey’s test, p>0.5). *Ae*. *simpsoni sl*. mosquitoes (4.8%, n = 2) developed midgut infection for NGIV on 7 dpi. However, no midgut infections were observed on days 14 and 21 post infection, hence we did not perform dissemination and transmission experiments (Fig 2A).

Bunyamwera virus disseminated infection was only observed on 7 and 21 dpi as opposed to 14 dpi in which no dissemination was recorded while disseminated infection was only recorded on 7 dpi for NGIV (Fig 2B). A total of 9 *Ae*. *simpsoni sl*. were tested for disseminated infection (2-NGIVand 7-BUNV). Overall, our results showed that 1 out of 2 of *Ae*. *simpsoni sl*. had disseminated infection for NGIV and 5 out of 7 for BUNV respectively. There was higher dissemination of BUNV observed between 7 and 21 dpi. *Ae*. *simpsoni sl*. had higher dissemination rate for BUNV than NGIV virus although the difference was not significant. The species had a dissemination of 4 out of 4 midgut infection on 7 dpi (Fig 2).

A total of 6 *Ae*. *simpsoni sl*. mosquitoes that had disseminated infections for NGIV (1) and BUNV (5) were tested for virus presence in the saliva. 1 out of 4 *Ae*. *simpsoni sl* with disseminated infection showed transmission for Bunyamwera on 7 dpi with no transmission was observed for NGIV (Fig 2B).

## Discussion

We evaluated the vector competence of a local population of *Ae*. *simpsoni sl*. from the coastal region of Kenya for BUNV and NGIV since implication of a particular mosquito species as a vector of a particular virus requires demonstration of mosquito vector competence and transmission in addition to detection of virus in field-collected mosquitoes [26]. The *Ae*. *Simpsoni sl* is an abundant species in the coastal area but the role of these species in transmission of NGIV and BUNV in the region remains unclear. Transmission of viruses largely depends on the potential of mosquitoes to acquire, maintain and disseminate the virus [27], in the study we have demonstrated that *Ae*. *Simpsoni sl* population is susceptible to midgut infection to both viruses, although the proportion of mosquitoes infected with these viruses was very low. All mosquito species tested ingested infected blood meal, but different barriers were observed per viruses with midgut barriers associated with low infection rates and midgut escape barrier associated with a small percentage of infected mosquitoes developing a disseminated infection as described by Turell et al [27]. The low midgut infection observed for both viruses is suggestive of the existence of a strong midgut infection barrier (MIB) [27, 28]. The low susceptibility to NGIV and BUNV infection in *Ae*. *simpsoni sl* population from Kilifi suggests both innate intrinsic and extrinsic factors that may contribute to mosquito resistance to virus infection [29], the same vector population has shown to be highly susceptible to chikungunya virus [23]. Mosquitoes’ immune responses and differences in the ecosystem may also influence the infection outcome of the mosquitoes and could be the reason for the observed low in infection rates [30]. Competent vectors must demonstrate ability of vector to be susceptible to infection by infecting the midgut barriers and be able to disseminate to the other parts of body and mostly important be able to transmit the virus [31] thus *Ae*. *simpsoni sl* is a competent vector for bunyawera virus.

Our study showed that there was dissemination of the NGIV and BUNV virus in *Ae*. *simpsoni sl*. at relatively higher rates for Bunyamwera than Ngari virus. Dissemination was recorded on day 7 for both BUNV and NGIV and, additionally, on 21 dpi for BUNV. High dissemination rate of BUNV indicates that the mosquitoes have a weak midgut escape barrier (MEB) for this virus than NGIV whose rates were low. Although this susceptibility may contribute to incrimination of a mosquito as a vector, other factors such as feeding preference and population must also be taken into account to implicate mosquito as a vector [32]. Transmission rates for BUNV were low and only occurred on 7 dpi, indicating a moderate midgut escape barrier (MEB) as described by Turell [33] as well as salivary gland infection (SGIB) and escape (SGEB) barriers. The MEB barrier was very strong for NGIV thus no dissemination was observed. Because of this, the strength of the SGIB and SGEB against NGIV remain undetermined. Future studies should consider using intrathoracic microinjections to better assess the permissiveness since oral exposure has been shown to have a strong midgut barrier for arboviruses.

## Conclusion

This study has shown the Coastal *Ae*. *simpsoni sl*. mosquito population are competent vectors of BUNV but susceptible to midgut infection and disseminated infection for NGIV. Although no transmission was observed for NGIV a disseminated infection in 1 out of 2 midgut-infected mosquitoes suggests possible transmission hence the vector may be competent bot both viruses. In light of the wide distribution of these mosquitoes in the coastal Kenya, incrimination of this species as a vector of the viruses presents a major threat to public health. Thus, a One Health approach to the promotion of understanding these viruses should be undertaken to define geographic risk regions, vector control strategies, and diagnostic development.

## Supporting information

S1 File (XLSX)

## References

[1] Bowen MD, Trappier SG, Sanchez AJ, Meyer RF, Goldsmith CS, Zaki SR, et al. A reassortant bunyavirus isolated from acute hemorrhagic fever cases in Kenya and Somalia. Virology. 2001;291(2):185–90. doi: 10.1006/viro.2001.1201 11878887

[2] Ellis BR, Wilcox BA. The ecological dimensions of vector-borne disease research and control. Cad Saude Publica. 2009;25(1):S155–67. doi: 10.1590/s0102-311x2009001300015 19287860

[3] Smithburn KC, Haddow AJ, Mahaffy AF. A neurotropic virus isolated from Aedes mosquitoes caught in the Semliki forest. The American journal of tropical medicine and hygiene. 1946;26:189–208. doi: 10.4269/ajtmh.1946.s1-26.189 21020339

[4] Wertheim HF, Horby P, Woodall JP. Atlas of human infectious diseases: John Wiley & Sons; 2012.

[5] Tauro LB, Rivarola ME, Lucca E, Mariño B, Mazzini R, Cardoso JF, et al. First isolation of Bunyamwera virus (Bunyaviridae family) from horses with neurological disease and an abortion in Argentina. Veterinary journal (London, England: 1997). 2015;206(1):111–4. doi: 10.1016/j.tvjl.2015.06.013 26183295

[6] Odhiambo C, Venter M, Chepkorir E, Mbaika S, Lutomiah J, Swanepoel R, et al. Vector Competence of Selected Mosquito Species in Kenya for Ngari and Bunyamwera Viruses. Journal of medical entomology. 2014;51(6):1248–53. doi: 10.1603/ME14063 26309314

[7] McIntosh BM, Worth CB, Kokernot RH. Isolation of Semliki Forest virus from Aedes (Aedimorphus) argenteopunctatus (Theobald) collected in Portuguese East Africa. Trans R Soc Trop Med Hyg. 1961;55:192–8. doi: 10.1016/0035-9203(61)90025-6 13774007

[8] Briese T, Bird B, Kapoor V, Nichol ST, Lipkin WI. Batai and Ngari viruses: M segment reassortment and association with severe febrile disease outbreaks in East Africa. Journal of virology. 2006;80(11):5627–30. doi: 10.1128/JVI.02448-05 16699043 PMC1472162

[9] Nashed NW, Olson JG, el-Tigani A. Isolation of Batai virus (Bunyaviridae:Bunyavirus) from the blood of suspected malaria patients in Sudan. The American journal of tropical medicine and hygiene. 1993;48(5):676–81. doi: 10.4269/ajtmh.1993.48.676 8517485

[10] Gerrard SR, Li L, Barrett AD, Nichol ST. Ngari virus is a Bunyamwera virus reassortant that can be associated with large outbreaks of hemorrhagic fever in Africa. Journal of virology. 2004;78(16):8922–6. doi: 10.1128/JVI.78.16.8922-8926.2004 15280501 PMC479050

[11] Zeller HG, Diallo M, Angel G, Traoré-Lamizana M, Thonnon J, Digoutte JP, et al. [Ngari virus (Bunyaviridae: Bunyavirus). First isolation from humans in Senegal, new mosquito vectors, its epidemiology]. Bulletin de la Societe de pathologie exotique (1990). 1996;89(1):12–6. 8765950

[12] Kokernot RH, Smithburn KC, De Meillon B, Paterson HE. Isolation of Bunyamwera virus from a naturally infected human being and further isolations from Aedes (Banksinella) circumluteolus theo. The American journal of tropical medicine and hygiene. 1958;7(6):579–84. doi: 10.4269/ajtmh.1958.7.579 13595198

[13] Rodrigues Hoffmann A, Dorniak P, Filant J, Dunlap KA, Bazer FW, de la Concha-Bermejillo A, et al. Ovine fetal immune response to Cache Valley virus infection. Journal of virology. 2013;87(10):5586–92. doi: 10.1128/JVI.01821-12 23468505 PMC3648191

[14] Jäckel S, Eiden M, El Mamy BO, Isselmou K, Vina-Rodriguez A, Doumbia B, et al. Molecular and serological studies on the Rift Valley fever outbreak in Mauritania in 2010. Transboundary and emerging diseases. 2013;60 Suppl 2:31–9. doi: 10.1111/tbed.12142 24589099

[15] Gordon SW, Tammariello RF, Linthicum KJ, Dohm DJ, Digoutte JP, Calvo-Wilson MA. Arbovirus isolations from mosquitoes collected during 1988 in the Senegal River basin. The American journal of tropical medicine and hygiene. 1992;47(6):742–8. doi: 10.4269/ajtmh.1992.47.742 1361722

[16] Yadav PD, Sudeep AB, Mishra AC, Mourya DT. Molecular characterization of Chittoor (Batai) virus isolates from India. Indian J Med Res. 2012;136(5):792–8. 23287126 PMC3573600

[17] Ochieng C, Lutomiah J, Makio A, Koka H, Chepkorir E, Yalwala S, et al. Mosquito-borne arbovirus surveillance at selected sites in diverse ecological zones of Kenya; 2007–2012. Virol J. 2013;10(140):10–140. doi: 10.1186/1743-422X-10-140 23663381 PMC3669043

[18] Sang R, Lutomiah J, Said M, Makio A, Koka H, Koskei E, et al. Effects of Irrigation and Rainfall on the Population Dynamics of Rift Valley Fever and Other Arbovirus Mosquito Vectors in the Epidemic-Prone Tana River County, Kenya. Journal of medical entomology. 2017;54(2):460–70. doi: 10.1093/jme/tjw206 28011732 PMC5850818

[19] Hardy JL, Houk EJ, Kramer LD, Reeves WC. Intrinsic factors affecting vector competence of mosquitoes for arboviruses. Annual review of entomology. 1983;28(1):229–62. doi: 10.1146/annurev.en.28.010183.001305 6131642

[20] Ciota AT, Kramer LD. Vector-virus interactions and transmission dynamics of West Nile virus. Viruses. 2013;5(12):3021–47. doi: 10.3390/v5123021 24351794 PMC3967159

[21] Mourya D, Yadav P, Mishra A. Effect of temperature stress on immature stages and susceptibility of Aedes aegypti mosquitoes to chikungunya virus. The American journal of tropical medicine and hygiene. 2004;70(4):346–50. 15100445

[22] Ellis BR, Sang RC, Horne KM, Higgs S, Wesson DM. Yellow fever virus susceptibility of two mosquito vectors from Kenya, East Africa. Trans R Soc Trop Med Hyg. 2012;106(6):387–9. doi: 10.1016/j.trstmh.2012.02.007 22521217

[23] Mulwa F, Lutomiah J, Chepkorir E, Okello S, Eyase F, Tigoi C, et al. Vector competence of Aedes bromeliae and Aedes vitattus mosquito populations from Kenya for chikungunya virus. PLoS neglected tropical diseases. 2018;12(10):e0006746. doi: 10.1371/journal.pntd.0006746 30321181 PMC6207330

[24] Camberlin P, Planchon O. Coastal precipitation regimes in Kenya. Geografiska Annaler: Series A, Physical Geography. 1997;79(1‐2):109–19.

[25] Jupp PG. Mosquitoes of Southern Africa: Culicinae and Toxorhynchitinae: Ekogilde Publishers; 1996.

[26] Reeves WC. Arthropods as vectors and reservoirs of animal pathogenic viruses. Handbuch der Virusforschung: Springer; 1958. p. 177–202.

[27] Turell MJ, Linthicum KJ, Patrican LA, Davies FG, Kairo A, Bailey CL. Vector competence of selected African mosquito (Diptera: Culicidae) species for Rift Valley fever virus. Journal of medical entomology. 2008;45(1):102–8. doi: 10.1603/0022-2585(2008)45[102:vcosam]2.0.co;2 18283949

[28] Turell MJ, Reeves WC, Hardy JL. Transovarial and trans-stadial transmission of California encephalitis virus in Aedes dorsalis and Aedes melanimon. The American journal of tropical medicine and hygiene. 1982;31(5):1021–9. doi: 10.4269/ajtmh.1982.31.1021 6889818

[29] Kramer LD, Ebel GD. Dynamics of flavivirus infection in mosquitoes. Advances in virus research. 2003;60:187–232. doi: 10.1016/s0065-3527(03)60006-0 14689695

[30] Houé V, Bonizzoni M, Failloux A-B. Endogenous non-retroviral elements in genomes of Aedes mosquitoes and vector competence. Emerging microbes & infections. 2019;8(1):542–55. doi: 10.1080/22221751.2019.1599302 30938223 PMC6455143

[31] Turell MJ, O’Guinn ML, Dohm DJ, Jones JW. Vector competence of North American mosquitoes (Diptera: Culicidae) for West Nile virus. Journal of medical entomology. 2001;38(2):130–4. doi: 10.1603/0022-2585-38.2.130 11296813

[32] Turell MJ, Reeves WC, Hardy JL. Evaluation of the efficiency of transovarial transmission of California encephalitis viral strains in Aedes dorsalis and Aedes melanimon. The American journal of tropical medicine and hygiene. 1982;31(2):382–8. doi: 10.4269/ajtmh.1982.31.382 7200334

[33] Turell MJ, Wilson WC, Bennett KE. Potential for North American mosquitoes (Diptera: Culicidae) to transmit rift valley fever virus. Journal of medical entomology. 2010;47(5):884–9. doi: 10.1603/me10007 20939385

